# Comparison of Human Hepatoma HepaRG Cells with Human and Rat Hepatocytes in Uptake Transport Assays in Order to Predict a Risk of Drug Induced Hepatotoxicity

**DOI:** 10.1371/journal.pone.0059432

**Published:** 2013-03-14

**Authors:** Monika Szabo, Zsuzsa Veres, Zsolt Baranyai, Ferenc Jakab, Katalin Jemnitz

**Affiliations:** 1 Institute of Molecular Pharmacology, Research Centre for Natural Sciences, Hungarian Academy of Sciences, Budapest, Hungary; 2 Department of Surgery, Uzsoki Teaching Hospital, Budapest, Hungary; Biological Research Centre of the Hungarian Academy of Sciences, Hungary

## Abstract

Human hepatocytes are the gold standard for toxicological studies but they have several drawbacks, like scarce availability, high inter-individual variability, a short lifetime, which limits their applicability. The aim of our investigations was to determine, whether HepaRG cells could replace human hepatocytes in uptake experiments for toxicity studies. HepaRG is a hepatoma cell line with most hepatic functions, including a considerable expression of uptake transporters in contrast to other hepatic immortalized cell lines. We compared the effect of cholestatic drugs (bosentan, cyclosporinA, troglitazone,) and bromosulfophthalein on the uptake of taurocholate and estrone-3-sulfate in human and rat hepatocytes and HepaRG cells. The substrate uptake was significantly slower in HepaRG cells than in human hepatocytes, still, in the presence of drugs we observed a concentration dependent decrease in uptake. In all cell types, the culture time had a significant impact not only on the uptake process but on the inhibitory effect of drugs too. The most significant drug effect was measured at 4 h after seeding. Our report is among the first concerning interactions of the uptake transporters in the HepaRG, at the functional level. Results of the present study clearly show that concerning the inhibition of taurocholate uptake by cholestatic drugs, HepaRG cells are closer to human hepatocytes than rat hepatocytes. In conclusion, we demonstrated that HepaRG cells may provide a suitable tool for hepatic uptake studies.

## Introduction

Drug-induced liver injury is one of the major reasons for the withdrawal of an approved drug from the market [1], [2]. These drugs show only a minor or no signs of hepatotoxicity in the animal species tested, indicating, that there is often poor correlation of toxicity from one species to another. Primary cultures of hepatocytes are the most common experimental system for studying *in vitro* drug metabolism and drug-transporter interactions [3], [4]. However, the use of human hepatocytes for toxicological studies has several drawbacks, such as their unpredictable and scarce availability, inter-individual variability, limited life span and phenotypic alterations [5]. These concerns have led to a call for alternative systems to screening and identifying potential toxic substances. Human immortalized liver cell lines could provide a solution to this problem. HepG2 and Fa2N-4 cells were the first alternatives but these cells have lost many liver-specific functions. In particular, expression levels of many cytochromes P450 and several hepatic sinusoidal transporters, including the uptake transporters were low or undetectable in these human cell lines [6], [7], [8]. All of these drawbacks limit the application of HepG2 and Fa2N-4 cells as an *in vitro* liver model for transport, metabolism and hepatotoxicity studies. HepaRG cell lines may be a potential tool for prediction of hepatotoxicity in preclinical drug development [9], [7]. HepaRG cells have been derived from a hepatocellular carcinoma cell line and can be differentiated from bi-potent progenitor cells to two distinct hepatic cell types, hepatocyte-like and biliary epithelial-like cells under a certain culture condition [10], [11], [12], [13]. Presently, only the HepaRG cells maintain several key hepatic functions, including metabolic enzymes, drug transporters and nuclear receptors at levels comparable with those found in primary human hepatocytes [9], [14], [7].

The aim of our investigations was to determine, whether HepaRG cells could replace human hepatocytes in toxicity studies and the preclinical screening of drug candidates. The present study mainly focussed on uptake processes; because we supposed that hepatotoxicity in humans may be associated with drug-mediated inhibition of uptake transporters [15], [16]. Many studies have demonstrated that uptake transporters are essential in the hepatic uptake of drugs from sinusoidal blood into the liver; therefore they play a crucial role in the drug elimination rate [17], [18]. Here we compared the inhibitory effect of drugs proved to be cholestatic during clinical use [19], [20], [21] and bromosulfophthalein (BSP) on the uptake of taurocholate (TC) and estrone-3-sulfate (E3S) in primary cultures of human, rat hepatocytes and HepaRG cells. TC is a typical substrate of sodium taurocholate cotrasporting polypeptide (NTCP/Ntcp) and some members of organic anion transporting polypeptide super family (OATPs/Oatps) are involved in the hepatic uptake of E3S in human (OATP1B1, OATP1B3 and OATP2B1) and rat (Oatp1a1, Oatp1a4 and Oatp1b2) hepatocytes [22].

## Methods

### Materials

^3^H-taurocholate (10 Ci/mmol) and ^3^H-estrone-3-sulfate (50 Ci/mmol) were obtained from American Radiolabeled Chemicals Inc (St Louis, MO). Bromosulfophthalein, cyclosporin A, estrone-3-sulfate, taurocholate, troglitazone, type IV collagenase, all cell culture media and reagents were purchased from Sigma-Aldrich (Budapest, Hungary). Bosentan was from Sequoia Research Products Ltd. (Pangbourne, UK). All other chemicals and reagents were of analytical grade and were readily available from commercial sources. Stock solutions of test compounds were prepared in dimethyl sulfoxide (DMSO). 24-well plates were obtained from Greiner Bio-One (Mosonmagyarovar, Hungary) or Biopredic International (Rennes, France). Sterile collagen from rat tail was prepared in-house according to established procedures.

### Conventional primary hepatocyte cultures

The isolation and conventional primary culture of rat hepatocytes were performed as described previously [23]. Hepatocytes from adult male Wistar rats (Charles River, Budapest) weighing 200 to 250 g were isolated by a previously described three-step collagenase perfusion method [23], [24]. The protocol was approved by the Institutional Animal Care and Use Committee (Research Centre of Natural Sciences, HAS, Budapest) and the Government Office for Pest County Food Chain Safety and Animal Health Directorate, Budapest, Hungary (Permit Number: 22.1/2728/3/2011). All surgery was performed under diethyl ether anesthesia, and all efforts were made to minimize suffering.

Human hepatocytes were prepared by perfusion of histologically normal liver fragments using a collagenase solution [23]. Human liver samples were obtained from adult donors undergoing hepatic resection at Uzsoki Hospital (Budapest, Hungary). All of the patients were operated with adenocarcinoma metastasis hepatis, and their age was 52–71, all of whom provided a written informed consent to participate in the study, which was approved by Scientific and Research Ethics Committee of the Medical Research Council (ETT-TUKEB ad.132/PI/2009-ad.8-47/2009-1018EKU), in adherence to the declaration of Helsinki.

Cell viability (>90%) was routinely checked by the trypan blue exclusion test. After preparation, freshly isolated cells were suspended in Williams' Medium E and plated at a cell density of 0.36×10^6^ cells per well in 24-well plates previously coated with rat tail collagen. The cells were initially grown in Williams' Medium E containing 5% of fetal calf serum (FCS), 100 nM insulin, 0.05 µM glucagon, 0.1 mg/ml gentamicin, 30 nM Na_2_SeO_3_, and 0.1 µM dexamethasone. The medium was discarded 24 h after seeding and hepatocytes were thereafter maintained in a serum-free medium supplemented with glucagon, insulin, gentamicin, dexamethasone, Na_2_SeO_3_ and renewed daily. Cells were maintained at 37°C in a humidified atmosphere of 95% air-5% CO_2_.

### Differentiated HepaRG cell cultures

The differentiated HepaRG (HPR116) cells were obtained from Biopredic International (Rennes, France). According to the supplier's review these cryopreserved cells are terminally differentiated and exhibit many characteristics of primary human hepatocytes including morphology, expression of key metabolic enzymes, nuclear receptors, and drug transporters. The fully differentiated HepaRG cultures seeded at high density contain about 50% of hepatocyte-like cells shortly after plating and later during culturing [10], [13]. HepaRG cells stored in liquid nitrogen were thawed according to the instructions of the supplier and resuspended in Basal medium for Thaw, Seed, and General Purpose HepaRG medium (Biopredic International). After checking viability the cells were seeded in collagen-coated 24-well plates (Biopredic International) at a cell density of 0.48×10^6^ cells/well. 24 h after plating the medium was changed to Basal medium for Maintenance (Biopredic International). The same procedure as that for hepatocytes was then followed.

### *In vitro* uptake experiments

For uptake experiments, HepaRG cells or/and hepatocytes were used at 4, 24 and 96 h after plating, respectively. The uptake assays were performed as described previously [25]. Briefly, the wells were washed once with 0.4 ml of Hanks' Balanced Salt Solution (HBSS). The uptake experiment was started by the addition of 0.4 ml of a HBSS solution containing the substrates (1 µM ^3^H-TC or ^3^H-E3S) and lasted for 30 sec (hepatocytes) or 300/120 sec with TC/E3S, respectively (HepaRG cells) at 37°C. The uptake was terminated by the removal of the substrate-containing buffer, and the wells were washed three times with ice-cold HBSS. Then the cells were lysed with 0.5 ml of 0.5% Triton X-100 solution. The intracellular radioactivity was determined by liquid scintillation counting. Protein content was determined by the method of Lowry et al. [26]. Assays were run using four wells in one set. All experiments were carried out with hepatocytes from three independent cell preparations.

### Drug interaction experiments

For drug interaction experiments, HepaRG cells were used at 4, 24 and 96 h, rat hepatocytes at 4, 24, 72 h and human hepatocytes 4, 24 and 120 h after plating, respectively. We investigated the inhibitory effect of drugs on the TC and E3S uptake as a function of drug concentration using the three cell types. The cells were incubated with 1 µM of ^3^H-TC or ^3^H-E3S in the presence of the inhibitors, bosentan (10–200 µM), bromosulfophthalein (BSP) (1–500 µM), cyclosporin A (CsA) (0.5–100 µM), troglitazone (5–100 µM), or the vehicle (DMSO, 0.1%), respectively. The uptake lasted for 30 sec (hepatocytes) or 300/120 sec, TC/E3S, respectively (HepaRG cells) at 37#C, and was terminated by the removal of the substrate and the modulator-containing medium. Then the cells were washed three times with ice-cold HBSS and lysed with 0.5 ml of 0.5% Triton X-100 solution. The intracellular concentrations of the substrates were determined by liquid scintillation counting. Assays were run using four wells as one set. All experiments were carried out with hepatocytes from three independent cell preparations.

### Statistics

The results are expressed as mean±SD for all experiments. Triplicate experiments of three independent hepatocyte isolations were run, and four wells were used for each set of conditions. IC_50_ values were calculated using OriginPro 8.5 statistical software.

## Results and Discussion

### Effect of culture time on TC and E3S uptake

**Figure 1:**
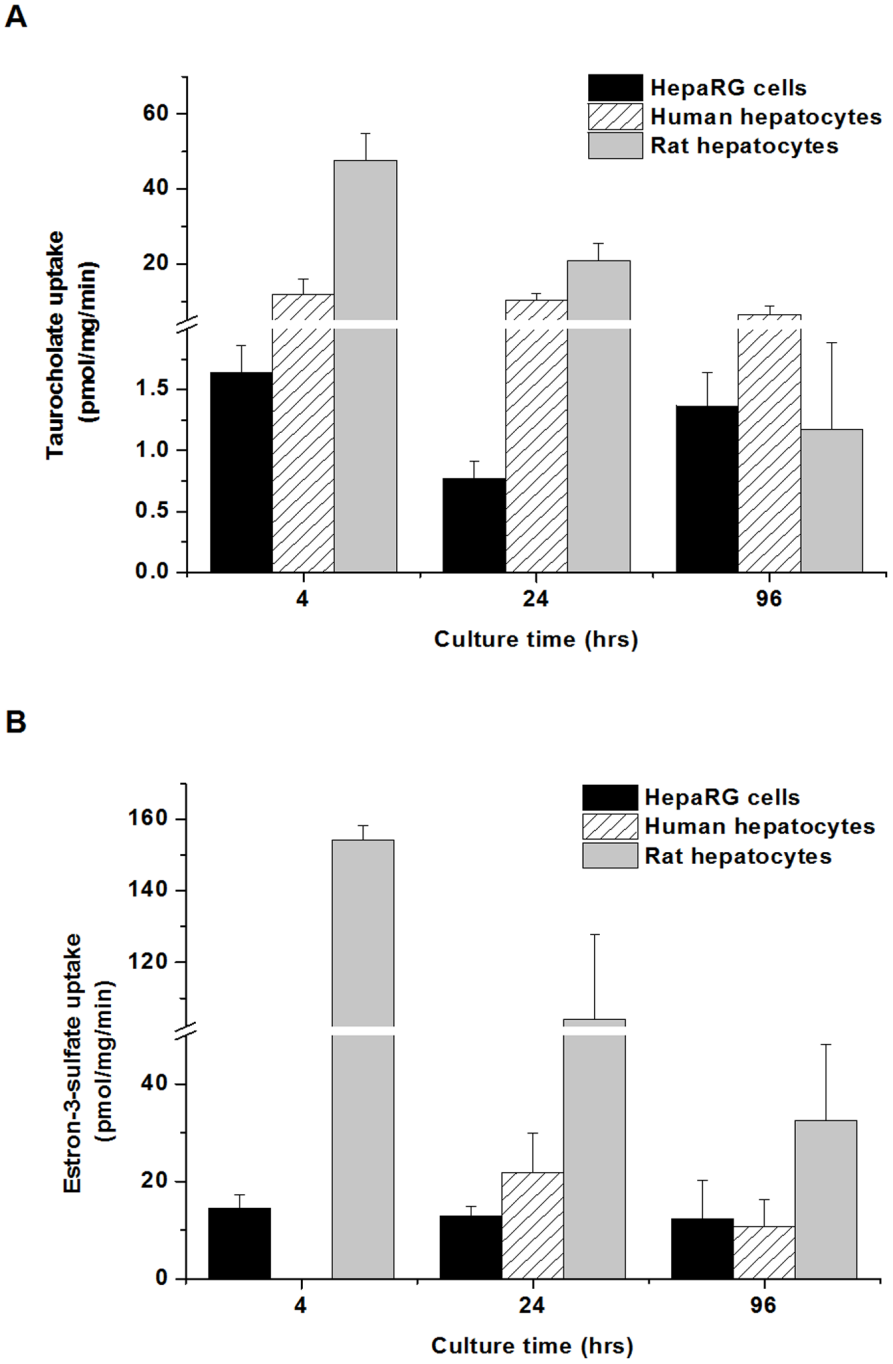
TC and E3S uptake in primary human and rat hepatocyte cultures and HepaRG cell cultures as a function of culture time. Cells were maintained in conventional monolayer culture. (A) Uptake of ^3^H-TC or (B) ^3^H-E3S (1µM) was measured for 30 sec (hepatocytes) or 300/120 sec, TC/E3S, respectively (HepaRG cells) at 4, 24 and 96 h after seeding. The intracellular amount of TC or E3S was determined from the lysates of the cells as described in Methods. Values are expressed as mean±SD, assays were run using four wells as one set. All experiments were carried out with hepatocytes from three independent preparations.

The effect of culture time on the uptake of TC and E3S was studied in conventional monolayer cultures of human and rat hepatocytes and HepaRG cells. The results are presented in Figure 1A and 1B. The uptake experiments lasted for 30 sec in case of primary hepatocytes, as we have already shown that the TC and E3S uptake is very rapid, especially at short after seeding [15]. In pilot studies, the uptake of TC and E3S was significantly slower in HepaRG cells compared to that in the primary hepatocytes, and was linear over a 5-min time period (data not shown). These findings are in accord with a previous study [7] demonstrating that expression of sinusoidal transporters such as NTCP and OATPs but OATP2B1 is significantly lower in HepaRG cells than in human hepatocytes. Therefore the incubation time of the uptake experiments was set at 5 min for TC and 2 min for E3S, respectively in case of HepaRG cell cultures.

**Figure 2:**
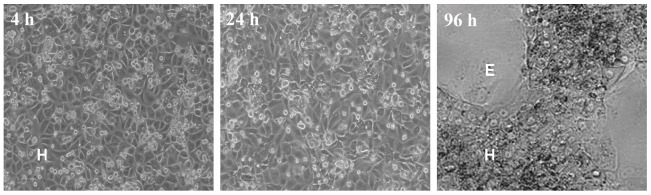
Phase-contrast image of HepaRG cells 4 h, 24 h and 96 h after seeding. H, hepatocyte-like cells; E, biliary epithelial-like cells.

Similarly to our previous results [15] the uptake of TC (Figure 1A) by rat hepatocytes measured at 4 h after seeding decreased to 44% during 24 h (from 47.7±7.3 pmol/mg/min to 20.9±4.7 pmol/mg/min), and drastically dropped (to 1.2±0.7 pmol/mg/min) in the course of longer culturing time (96 h). This observation is in accord with the findings of Rippin et al. [27], who showed that in conventional monolayer culture of rat hepatocytes the mRNA and protein levels of Ntcp and Oatp1a1 decreased to a few percent of the initial level by 72 h. On the contrary, TC uptake activities remained relatively high in human hepatocytes in the first 24 h after seeding (11.9±4.2 pmol/mg/min and 10.4±1.8 pmol/mg/min), and slightly diminished (6.6±2.1 pmol/mg/min) over time in culture. Our results are confirmed by the work of Jigorel et al. [28], who demonstrated that the mRNA level of NTCP was better maintained in a 3-day-old human culture than in their rat counterparts. In HepaRG cells the TC uptake significantly decreased by 24 h in culture (from 1.6±0.2 pmol/mg/min to 0.7±0.1 pmol/mg/min) but returned to almost the initial value by 96 h (1.3±0.2 pmol/mg/min). This could be related to the fact that within a day following thaw and seeding transporter activities decrease while the cells reconstitute the monolayer, then the activities return similarly to CYP activities [10]. The appearance of two, morphologically different cell types in a 96 h HepaRG cell culture is demonstrated in Figure 2. A 7.2-fold lower TC uptake was measured at short after seeding in HepaRG cells compared to that observed in human hepatocytes, in agreement with the findings of Le Vee et al [7].

E3S uptake (Figure 1B) significantly decreased in rat hepatocytes during culturing (from 154.1±4.0 pmol/mg/min to 32.6±15.6 pmol/mg/min by 96 h); however, the decrease was not as substantial as in the case of TC. Our data support that Oatps are better maintained than the Ntcp in rat hepatocytes during culturing. Human hepatocytes showed a smaller decrease of E3S uptake compared with rat hepatocytes (from 21.9±8.0 pmol/mg/min to 10.8±5.5 pmol/mg/min, from 24 h to 96 h). These observations are consistent with the report of Jigorel et al. [28], who described better maintained Oatps/OATPs activities in both rat and human hepatocyte cultures. In HepaRG cells, E3S uptake did not change significantly, with time in culture. Approximately a 3-fold lower E3S uptake was measured in a 96-hour-old HepaRG and human hepatocyte culture than in rat hepatocytes.

### Effect of Bosentan and CsA on TC uptake

The inhibition of normal bile acid transport is one of the possible mechanisms causing hepatotoxicity [20]. In this work, we focussed primarily on compounds that were shown to modulate bile salt homeostasis mostly by BSEP inhibition [29]. Kostrubsky et al. [30] reported that bosentan and CsA inhibit bile acid biliary excretion in sandwich-cultured human hepatocytes. Although it has been shown that some of these xenobiotics may alter bile acid homeostasis by inhibiting both hepatic uptake and canalicular efflux of them [15], [31], the effect of these drugs using HepaRG cell line as a model has not been investigated yet. TC, an endogenous compound, is commonly used as a model substrate for bile acid transport studies. It is well published that the hepatic uptake of TC is predominantly mediated by NTCP/Ntcp [32], [33], which is expressed exclusively in the liver. Human NTCP has a higher affinity towards TC than rat Ntcp (K_m_ of 6 and 25 µM, respectively) [22]. It is worth mentioning that most of the drugs that have been proved to inhibit bile salt uptake are not transported by NTCP/Ntcp.

**Figure 3:**
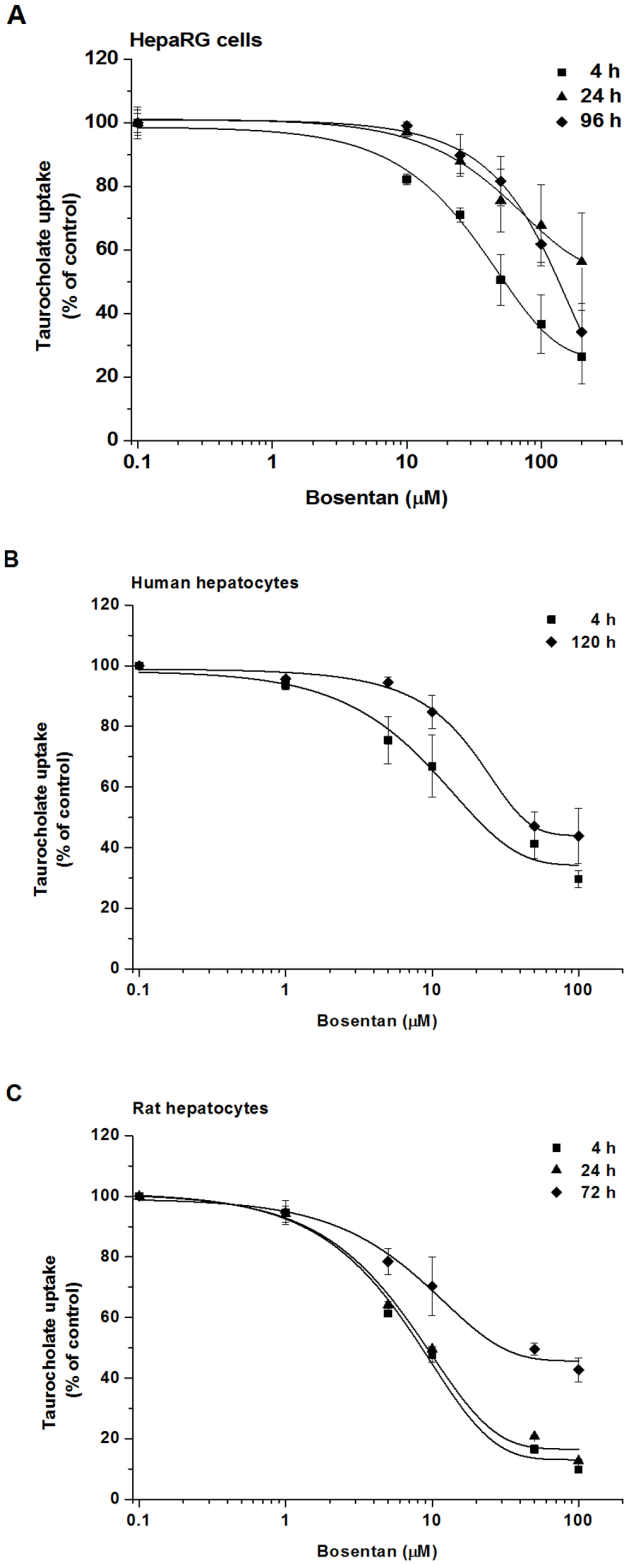
Comparison of the inhibition of TC uptake by bosentan in human and rat hepatocyte cultures and HepaRG cell cultures. (A) HepaRG cells were treated with 1 µM of ^3^H-TC for 300 sec at 4, 24 and 96 h after seeding in the presence of bosentan (10–200 µM), or the vehicle, respectively. (B) Human and (C) rat hepatocytes were treated with 1 µM of ^3^H-TC for 30 sec at 4, 24 and 72 h (rat) or 4, 120 h (human) after seeding in the presence of bosentan (10–100 µM), or the vehicle, respectively. Values are expressed as mean±SD, assays were run using four wells as one set. All experiments were carried out with hepatocytes from three independent preparations.

Figure 3A, 3B and 3C show the concentration-dependent inhibition of TC uptake by bosentan in the three cell types. Bosentan, which is a substrate for OATP1B1 and OATP1B3 and not transported by NTCP [18], decreased the TC uptake with an approximately 3-fold lower IC_50_ in freshly seeded rat than human hepatocytes (IC_50_ of 8.9 and 28.4 µM respectively) (Table 1). Our results are in accord with the findings of Leslie et al. [31], who described an IC_50_ of 5.4 µM and 30 µM for bosentan in primary rat and human hepatocyte suspensions, respectively. However, this difference between the IC_50_ values obtained with the two cell types vanished in time, suggesting that the expression of hepatic uptake transporters is better preserved in primary human than in rat hepatocytes. In HepaRG cells at 4 h after seeding bosentan inhibited TC uptake with an approximately 2-fold higher IC_50_ than in human hepatocytes (Table 1). In a 24 h HepaRG culture the decrease of TC uptake was not more than 40% of the control even at 200 µM bosentan concentration. By 96 h, the inhibition potential of bosentan increased, but did not reach the effectiveness observed at 4 h after seeding (Table 1). These findings are in close relation with the results of the uptake studies. A declining TC uptake observed in all cell types was probably due to the reduced expression of NTCP/Ntcp, which resulted in a loss of the inhibition capacity of drugs. In contrast to the primary hepatocytes HepaRG cells regained part of their TC uptake capacity during culturing, which led to the increase of drug effect. It is worth mentioning that in cholangiocyte-like cells the apical sodium-dependent bile salt transporter (ASBT/SLC10A2), which is involved in the enterohepatic circulation of bile salts, might have a different affinity towards the drugs studied, and may contribute to the inhibition of TC uptake in the HepaRG cell cultures at 96 h [34], [10].

**Table 1:** Inhibitory potential of drugs towards TC uptake.

Compound	IC_50_ values (µM)								
	HepaRG cells			Human hepatocytes			Rat hepatocytes		
	4 h	24 h	96 h	4 h	24 h	120 h	4 h	24 h	72 h
Bosentan	50.9	>200	137.5	28.4	nt^a^	44.8	8.9	9.7	48.4
Cyclosporin A	1.2	>50	4.7	2.4	5.0	>10	3.9	11.8	>100
Troglitazone	8.4	29.5	10.3	nt	nt	nt	nt	nt	nt

a nt = not tested.

Data are presented as mean of three independent cell preparations.

**Figure 4:**
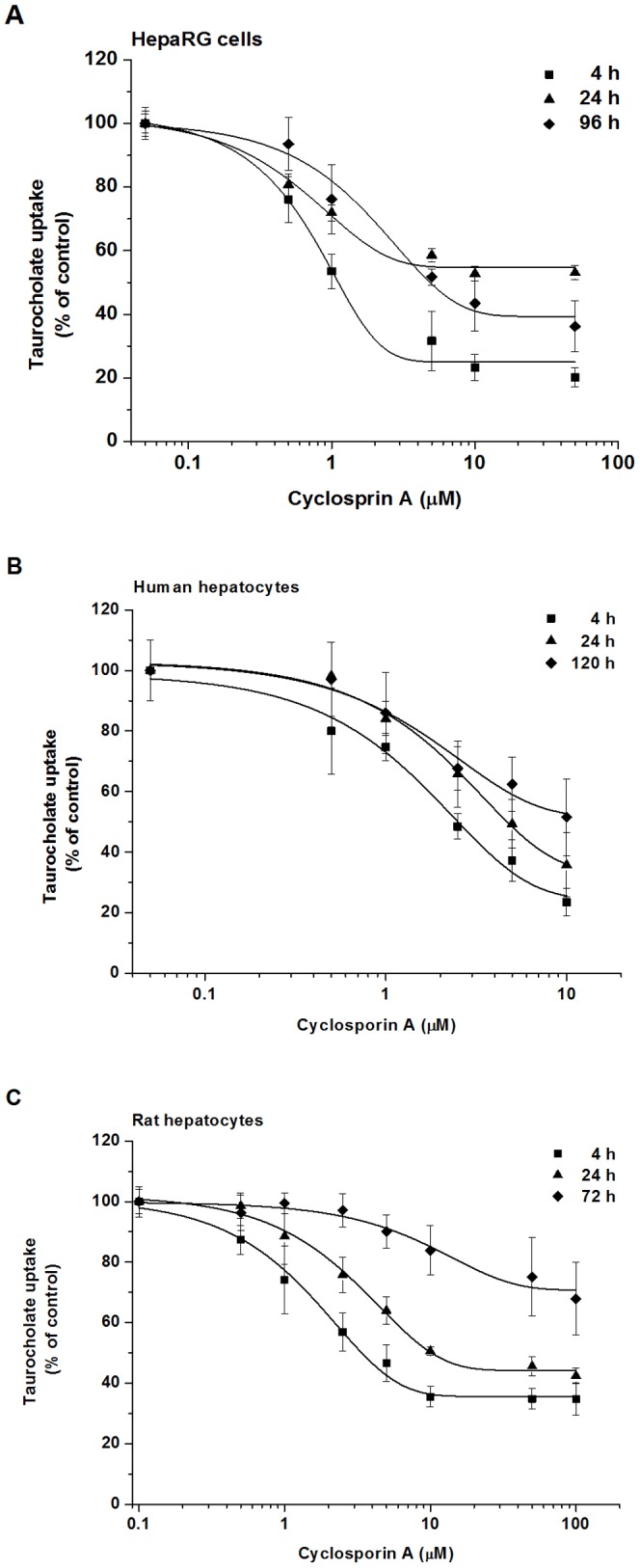
Comparison of the inhibition of TC uptake by cyclosporin A in human and rat hepatocyte cultures and HepaRG cell cultures. (A) HepaRG cells were treated with 1 µM of ^3^H-TC for 300 sec at 4, 24 and 96 h after seeding in the presence of cyclosporin A (0.5–50 µM), or the vehicle, respectively. (B) Human and (C) rat hepatocytes were treated with 1 µM of ^3^H-TC for 30 sec at 4, 24 and 120 h (human) or 4, 24 and 72 h (rat) after seeding in the presence of cyclosporin A (0.5–10 µM or 0.5–100 µM), or the vehicle, respectively. Values are expressed as mean±SD, assays were run using four wells as one set. All experiments were carried out with hepatocytes from three independent preparations.

Figure 4A, 4B and 4C show the decrease in TC uptake as a function of CsA concentration in human and rat hepatocytes and HepaRG cells. The extent of inhibition was similar in HepaRG cells and human hepatocytes, and it was less pronounced in rat cells at 4 h after seeding. This observation is consistent with previous reports showing that CsA inhibited human NTCP more efficiently than rat Ntcp [35], [36]. The inhibitory effect of CsA was similar in 24 h human hepatocytes and 96 h HepaRG cells (IC_50_ of 5.0 and 4.7 µM respectively) (Table 1). The inhibitory potential of CsA, just like that of bosentan, dramatically decreased in a 3-day-old rat hepatocyte culture. CsA proved to be a more potent inhibitor than bosentan in all cell types. CsA is able to inhibit both sodium-dependent (NTCP) and sodium-independent (OATP1B1, OATP1B3) TC uptake into human hepatocytes, which may contribute to its higher interaction potential observed [37], [18].

### Effect of Troglitazone on TC uptake

**Figure 5:**
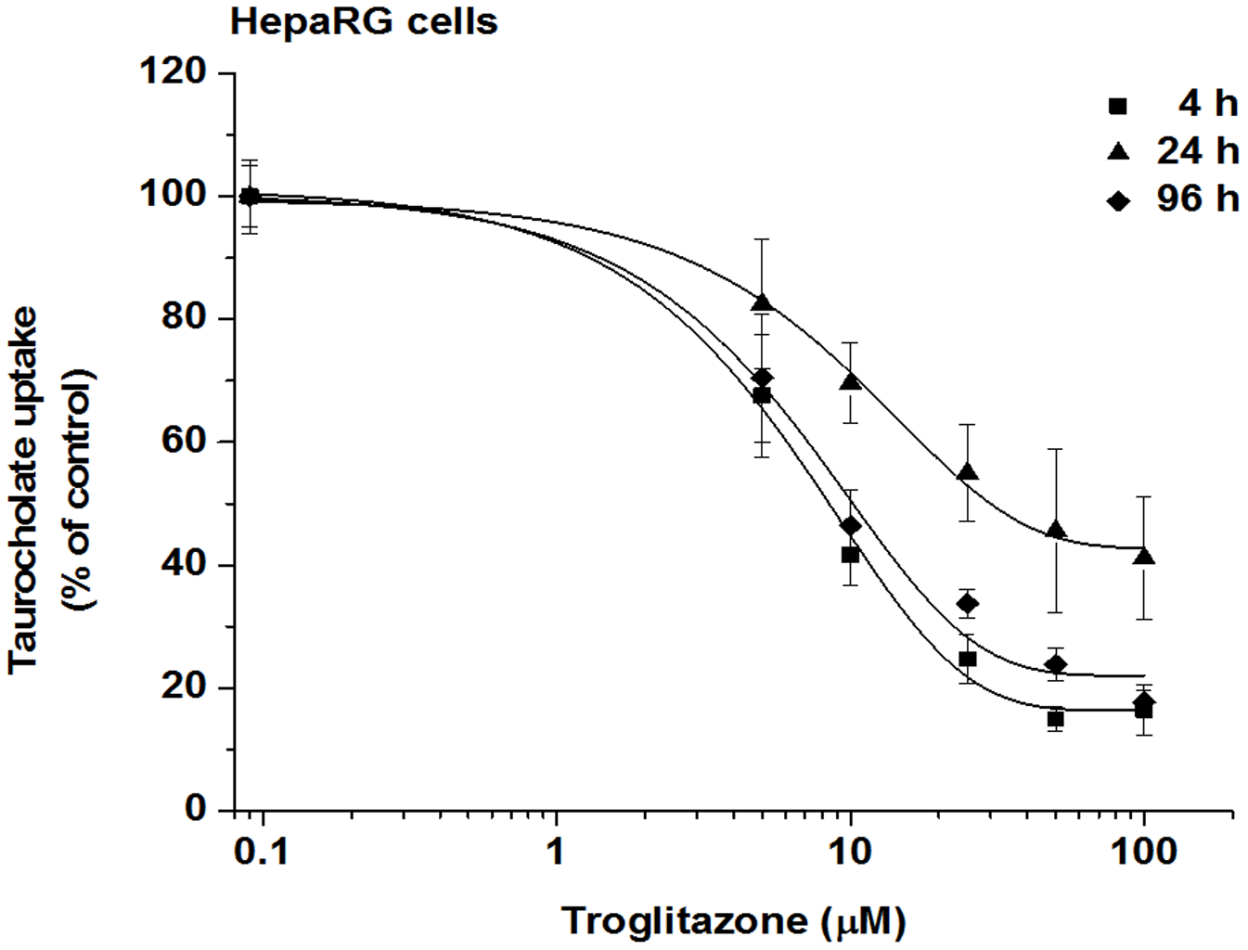
Inhibition of TC uptake by troglitazone in HepaRG cells. HepaRG cells were treated with 1 µM of ^3^H-TC for 300 sec at 4, 24 and 96 h after seeding in the presence of troglitazone (5–100 µM), or the vehicle. Values are expressed as mean±SD, assays were run using four wells as one set.

Troglitazone was removed from US markets in 2000 because its use was associated with liver failure [38]. Funk et al. [21] demonstrated that troglitazone inhibits Bsep-mediated TC biliary excretion in a concentration-dependent manner in rat hepatocytes. We investigated whether this drug decreases the TC uptake also in HepaRG cells. The concentration dependent effect of troglitazone on TC uptake by HepaRG cells is shown in Figure 5. Troglitazone inhibited the uptake of TC with almost the same IC_50_ (8.4 and 10.3 µM) at 4 and 96 h (Table 1). IC_50_ measured at 24 h was about 3-fold higher than that at 4 h and 96 h supporting the regained NTCP expression by 96 h. Marion et al. [12] examined the effect of troglitazone on the transport of TC in human and rat hepatocyte suspension and demonstrated that troglitazone was a more potent inhibitor for human NTCP (IC_50_ of 0.33 µM) than for rat Ntcp (IC_50_ of 2.3 µM). The difference between NTCP versus Ntcp inhibition could be one reason for species differences regarding drug-induced hepatotoxicity.

Preliminary viability studies (MTT assay) confirmed that the cholestatic drugs applied in the uptake experiments were not cytotoxic for either cell type even at the highest concentration used (data not shown).

### Effect of BSP on E3S uptake

**Figure 6:**
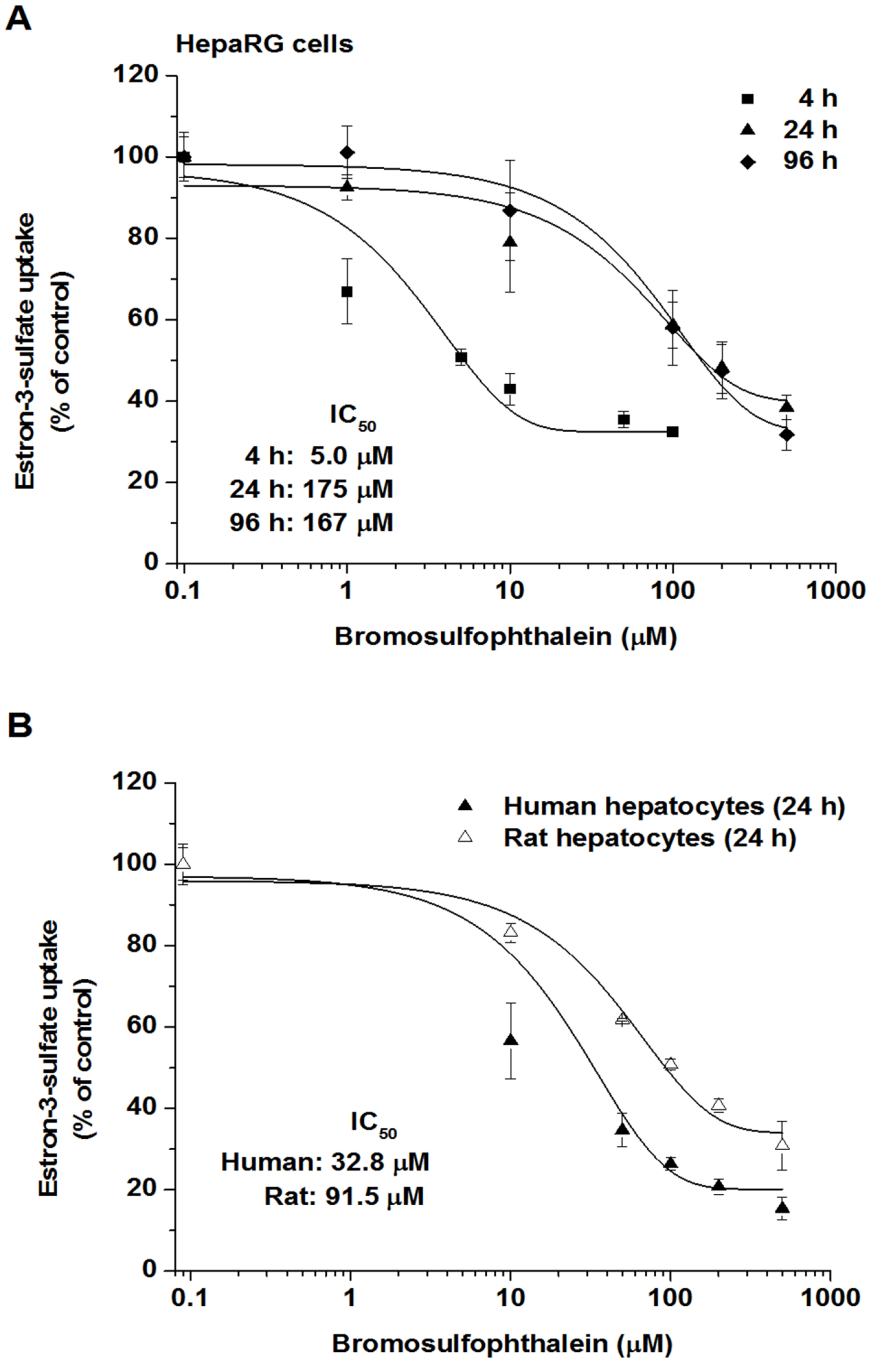
Comparison of the inhibition of E3S uptake by bromosulfophthalein in human and rat hepatocytes and HepaRG cells. (A) HepaRG cells were treated with 1 µM of ^3^H-E3S for 120 sec at 4, 24 and 96 h after seeding in the presence of bromosulfophthalein (1–500 µM), or the vehicle. (B) Human and rat hepatocytes were treated with 1 µM of ^3^H-E3S for 30 sec at 24 h after seeding in the presence of bromosulfophthalein (1–500 µM), or the vehicle. Values are expressed as mean±SD, assays were run using four wells as one set. All experiments were carried out with hepatocytes from three independent preparations.

Furthermore, we also examined the interaction potential of BSP concerning the E3S uptake in the cell cultures (Figure 6A and 6B). As shown in Figure 6B, BSP inhibited the E3S uptake with a 2.8-fold lower IC_50_ in human than in rat hepatocytes at 24 h after seeding. In HepaRG cells BSP decreased the uptake of E3S with an IC_50_ of 5.0 µM at 4 h after seeding (Figure 6A). Although the uptake rate of E3S was constant during culturing, the inhibitory effect of BSP decreased by 24 h and did not change thereafter. This inhibitory pattern of BSP seems to be different from that found in the case of drugs influencing TC uptake, where the IC_50_ values were always much lower at 96 than at 24 hours after seeding. Further studies are needed to estimate the expression level of different OATPs involved in the uptake of E3S as a function of culture time and cell cycle in HepaRG cells in order to elucidate the inhibition pattern obtained for BSP. In a recent paper Kotani et al. [39] studied the inhibition potencies of 12 OATP1B1 inhibitors both in HepaRG cells and in OATP1B1-expressing HEK293 cells and demonstrated the usefulness of HepaRG cells for the characterization of OATP1B1-mediated drug-drug interactions.

In conclusion, we demonstrated the applicability of HepaRG cell line as a potential alternative to primary human hepatocytes in an uptake model and suggest that HepaRG cells can help predict drug related hepatotoxicity in humans. So far, this is the first report in which drug interaction with taurocholate and estrone-3-sulfate uptake transport was presented in an immortalized cell line. We demonstrated that concerning the inhibition of TC uptake process, HepaRG cells are closer to human hepatocytes than rat hepatocytes. It should be kept in mind that the expression levels of sinusoidal drug transporters are lower in HepaRG cells than in primary hepatocyte cultures, so longer incubation times are needed [7]. And it is noteworthy that culture time is a critical parameter for drug interaction, which corresponds with our findings in our previous report [15]. A shorter culture time provides more reliable results of drug interaction potential with uptake transporters. Taking all together, our results suggest that HepaRG cell line may be a suitable model to study not only metabolic processes but uptake transport as well.
